# Recent advances in experimental and clinical applications of chest electrical impedance tomography: a narrative review

**DOI:** 10.1186/s40635-025-00848-3

**Published:** 2026-01-06

**Authors:** Inéz Frerichs, Gaetano Scaramuzzo, Annemijn Jonkman

**Affiliations:** 1 Department of Anaesthesiology and Intensive Care Medicine, University Medical Centre Schleswig-Holstein, Campus Kiel, Arnold-Heller-Strasse 3, 24105 Kiel, Germany; 2 Department of Translational Medicine, University of Ferrara, Ferrara, Italy; 3 Intensive Care Unit, Department of Emergency, Sant’Anna University Hospital, Ferrara, Italy; 4 Department of Adult Intensive Care, Erasmus Medical Centre, Rotterdam, The Netherlands

**Keywords:** EIT, Functional lung imaging, Mechanical ventilation, Ventilator settings

## Abstract

The achieved technological maturity of electrical impedance tomography (EIT) and the clinical need of the information provided by this functional imaging method has intensified research activities on the medical use of chest EIT. The recent years have witnessed an accelerated research covering not only the experimental setting but also the clinical environment with the major focus on mechanically ventilated patients, both in the perioperative period or as part of the intensive care treatment. Patients of all age groups are being included in clinical investigations and studies using EIT. The major objectives for use of EIT are the monitoring of regional lung and cardiovascular function, identification of adverse events (pneumothorax, alveolar overdistension and collapse, pulmonary embolism) and guidance for individualised therapy (selection of ventilator setting, positioning and physical therapy). Our review describes the most recent achievements of experimental and clinical research on chest EIT. The provided information helps to identify the current hot topics in EIT research and to guide further improvements of EIT technology and applications that are still needed to enforce the establishment of chest EIT in routine patient care.

## Introduction

The intensive care medicine community has witnessed an accelerated research on chest electrical impedance tomography (EIT) in the recent years. This is evidenced by an increasing number of publications reporting the results of both experimental and clinical applications of chest EIT. The observed boost in EIT-related research has been facilitated by two major factors. One of them is the technological advancement of EIT, offering reliable and approved medical devices for bedside use. The other is the clinical need of the information generated by EIT. Chest EIT is primarily able to monitor the distribution of regional lung ventilation and aeration. This information is of relevance especially in mechanically ventilated patients where it could be utilised for guiding individualised ventilator therapy.

We discuss the most recent achievements in chest EIT research, covering the period from January 2024 till August 2025. The aim was to identify the current hot topics in chest EIT research, generate a structured summary of both experimental and clinical studies and provide a basis for planning of focussed EIT advancements. Our review does not contain information on EIT basics and the general principles of use which can be found in other sources [1, 2].

The review is organised in sections, covering the experimental applications of chest EIT, clinical perioperative and ICU applications in adult patients, the specific use in neonatal and paediatric patients and methodological advancements.

## Experimental applications

Experimental EIT applications have been crucial for development of the technique before reaching bedside implementation, and to enhance our understanding of pulmonary physiology and the impact of mechanical ventilation settings.

**Fig. 1:**
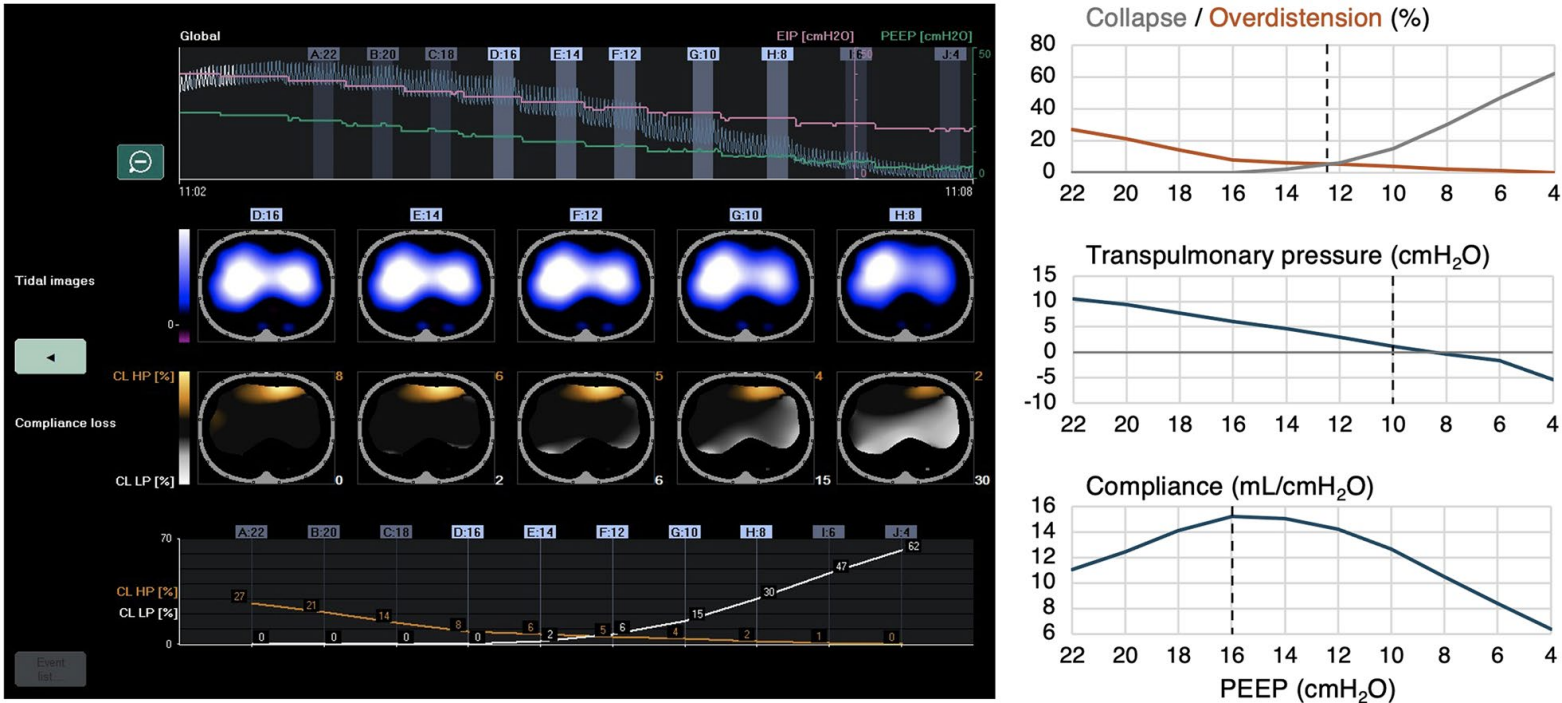
EIT-based PEEP titration compared to methods derived from transpulmonary pressure and respiratory system compliance measurements. The left panel shows (1) the global EIT, end-inspiratory pressure (EIP, purple line) and positive end-expiratory pressure (PEEP, green line) waveforms recorded in a porcine lung injury model during a decremental PEEP trial (top), (2) functional EIT images presenting the ventilated lung regions (blue-white) and regions of overdistension (orange brown) and collapse (light grey) for the five highlighted PEEP steps (middle) and (3) the overdistension and collapse curves originating from the values of compliance loss toward higher PEEP (CL HP, orange brown line) and lower PEEP (CL LP, light grey line). The right panel illustrates that the crossing-point PEEP level differs from that as suggested by the transpulmonary pressure method targeting a positive end-expiratory value, or when titrating the highest respiratory system compliance. Representative case example kindly provided by Dr. Sousa, from his study [4]

In the adult ICU, the main use-case of EIT is setting PEEP but the optimal approach and impact of EIT-based strategies on respiratory mechanics and cardiovascular function are less well understood. In a porcine model of highly recruitable ARDS, Sousa et al. [3] compared three different EIT-based PEEP setting strategies for 12 h, including the crossing point of overdistension and collapse (OD–CL), or targeting low (≤3%) overdistension or low collapse, while ventilating pigs lung-protective with low tidal volumes (*V*_T_). In the low overdistension group, accepting high amounts of collapse (and shunting), 50% of pigs died prior to experiment completion due to cardiopulmonary dysfunction. In contrast, the OD–CL and low collapse strategies resulted in better gas exchange and mechanics, with more homogeneous lungs with the OD–CL approach. In a secondary analysis, Sousa et al. [4] compared the OD–CL approach to non-EIT guided PEEP setting (including highest respiratory system compliance (*C*_rs_) and positive end-expiratory transpulmonary pressure (*P*_L_)) (Fig. 1). Different methods resulted in different suggested PEEP levels, with the EIT approach generally resulting in lower PEEP compared to the *C*_rs_ method, but higher than the *P*_L_ approach; between-method differences were more pronounced in bilateral than unilateral lung injury.

In a swine model of lung atelectasis, Vivona et al. [5] evaluated the effect of conventional recruitment manoeuvres and variable ventilation with breath-by-breath variation of *V*_T_ during controlled modes on ventilation and perfusion. Both methods successfully recruited the lungs (on CT scan), and the centre of ventilation and perfusion slightly shifted to the dorsal regions on EIT. Recruitment manoeuvres negatively affected haemodynamics which was prevented with variable ventilation.

Beyond ventilator pressures, changing body positioning could recruit the lungs, which can be guided by EIT monitoring. Mlček et al. [6] performed a sequential lateral position strategy in a 2-hit ARDS porcine model while maintaining sufficient PEEP. EIT demonstrated that regional compliance and ventilation of the dorsal lung greatly improved along the lateral positioning strategy, without causing overdistension. Perfusion also shifted to the nondependent lung and oxygenation improved.

When setting PEEP, overcoming the airway opening pressure (AOP) might prevent repeated airway closure/re-opening (atelectrauma). AOP can be measured on the ventilator (PV loops or pressure–time curve) during a low-flow inflation manoeuvre, whereas EIT can identify regional AOP [7]. In a swine model of severe ARDS, Pellegrini et al. [8] monitored regional ventilation and perfusion when PEEP was set above or below AOP (confirmed by EIT), and tested the effect of inhaled nitric oxide (iNO) in both conditions. They found that iNO efficacy was influenced by the PEEP level relative to AOP: in bilateral injury, iNO improved regional ventilation/perfusion ($\dot{V}/Q$) matching only when PEEP was set above AOP. In asymmetrical injury this effect existed independent of the PEEP set. Clinical implications are that the distribution of lung injury and recruitability play a role in iNO efficacy—both can be monitored with EIT.

### Lung perfusion and $\dot{V}/Q$ matching

Although perfusion and $\dot{V}/Q$ measurement with EIT are less evolved than ventilation monitoring, there have been new experimental developments in this context. The bolus technique using hypertonic saline has been the standard for EIT perfusion assessment, where perfusion is derived through kinetic analysis of the conductive agent passage through pulmonary vessels.

As noninvasive alternative, perfusion has also been studied through the heartbeat-related pulsatile EIT signal. In a pig model with acute pulmonary embolism, Li et al. [9] compared this method to the bolus technique and found comparable results in terms of $\dot{V}/Q$ match at different pulmonary perfusion stages. However, as both techniques are based on fundamentally different principles, a reliable direct comparison of perfusion-derived parameters is impossible.

In an attempt to derive more meaningful information from the EIT perfusion image, $\dot{V}/Q$ parameters can be calibrated. Calibration of ventilation impedance changes to minute ventilation is easy when ventilator parameters are known, but calibrating the perfusion-related impedance changes is more challenging as it requires monitoring of cardiac output (CO). Calibrating $\dot{V}/Q$ to *V*_T_/CO was described previously [10] and Han et al. [11] recently evaluated a new approach utilising the arterial blood pressure area under the curve (AUC). They found comparable results between the $\dot{V}/Q$ calibration methods in pigs without lung injury, after pulmonary embolism and atelectasis.

### EIT during spontaneous breathing

EIT has been used to study the impact of excessive breathing efforts on regional lung mechanics in the context of patient self-induced lung injury mechanisms [12]. There has been special interest in measuring the pendelluft, which is the pendulum-like air displacement between different lung regions increasing regional lung stress. EIT is the only bedside method to quantify this phenomenon and its first description dates back to 2013 [13]. A recent study by Wittenstein et al. [14] in an ARDS porcine model indicated that asynchronous breathing efforts resulted in pendelluft more frequently than synchronised efforts; however, they found no correlations between asynchrony and lung injury and diaphragm dysfunction.

## Clinical applications in a perioperative setting

EIT can be useful throughout the different phases of the surgical process: (1) in the preoperative phase for assessing baseline ventilation patterns, (2) during surgery for PEEP titration, recruitment assessment and ventilation strategy optimisation which is crucial for preventing postoperative pulmonary complications (PPCs), and (3) after surgery, for patient phenotyping and prediction or potential diagnosis of respiratory complications.

The recent EIT applications in the perioperative setting are summarised below.

### EIT before surgery

Although EIT is primarily used intraoperatively, some studies have suggested its preoperative use for screening ventilatory patterns, aiming to identify patients with nonhomogeneous distribution or areas of hypoventilation that may be more vulnerable to the development of atelectasis. For example, observational studies have shown that subjects with reduced dorsal ventilation at baseline are more likely to experience alveolar collapse immediately after induction [15], while other studies have dynamically shown the regional formation of atelectasis, especially in the obese population [16]. Some case reports identified a possible EIT application in the evaluation of peri-induction complications, such as pulmonary aspiration [17]. These observations suggest a role for EIT in the prompt evaluation of anaesthesia-related complications. However, randomised trials demonstrating impact on clinical practice during routine monitoring are lacking.

### EIT during surgery

#### PEEP titration

**Fig. 2:**
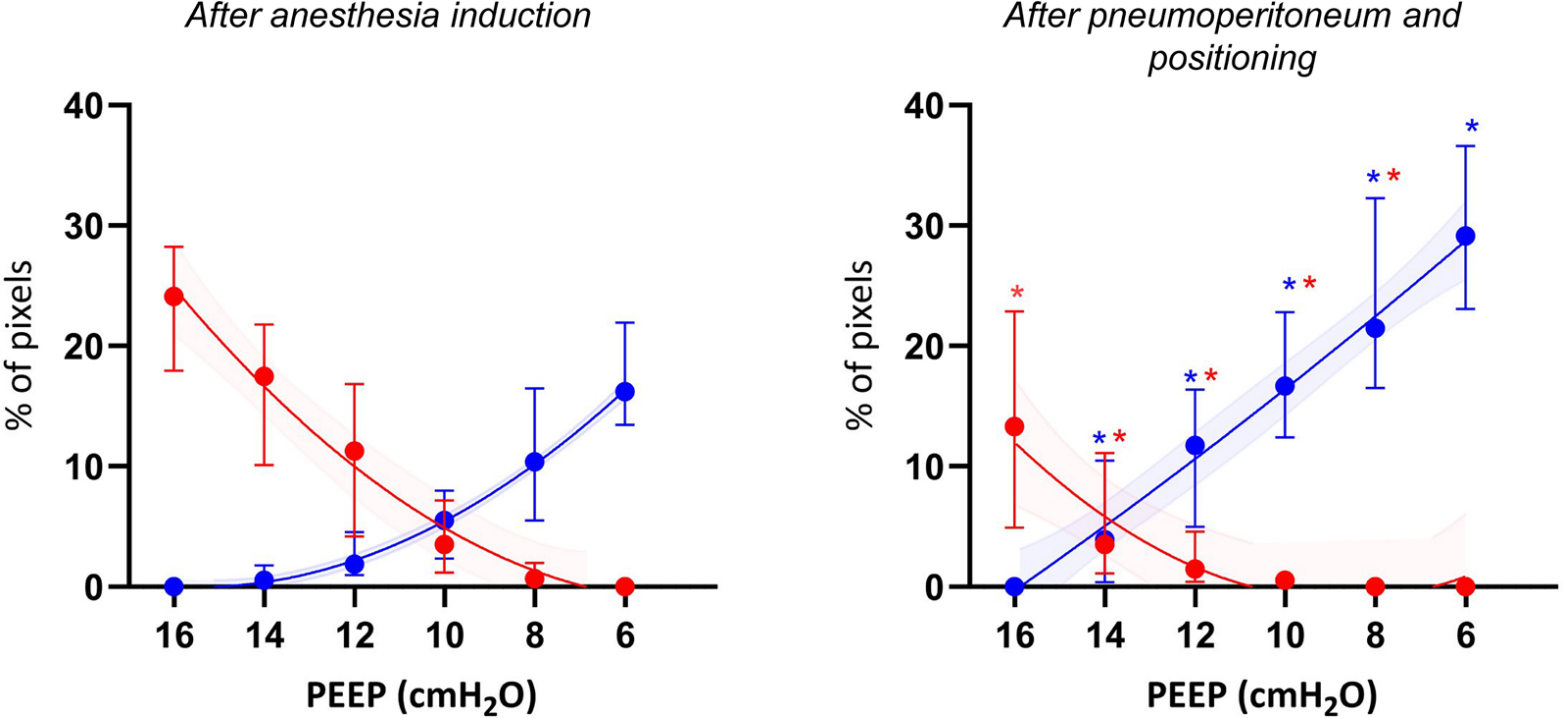
EIT-based PEEP titration in laparoscopic surgery. Relative collapse and overdistention curves were determined by EIT during a PEEP titration trial from 16 to 6 cmH_2_O. Percentage of collapsed (blue) and overdistended (red) pixels are shown after intubation (left) and pneumoperitoneum and patient positioning (right). * Significant difference at *p* ≤ 0.05. The figure is reproduced under a Creative Commons Attribution 4.0 International License from [19]

EIT-based PEEP adjustment is the most widely explored area in the surgical patient, using the OD–CL method that was first described in the ARDS setting [18]. Figure 2 illustrates the findings obtained by this method.

Several randomised trials and physiological studies have shown that EIT-based PEEP titration improves ventilation distribution, reduces atelectasis formation and limits hyperinflation. An early study on patients undergoing laparoscopic and open abdomen surgery [20] showed that PEEP setting using EIT reduced postoperative atelectasis and intraoperative driving pressure compared to the standard of care (fixed PEEP). Ma et al. [21] and Pan et al. [22] confirmed this in other surgical settings, as individualised PEEP using EIT enhanced respiratory mechanics, reduced the incidence of post-operative atelectasis and led to better oxygenation compared to fixed PEEP.

In a recent trial on obese patients undergoing laparoscopic surgery [19], EIT showed that the optimal PEEP setting varied during the procedure, suggesting the necessity for continuous monitoring. Similar findings supporting the concept of a dynamic tailored PEEP strategy, adapting the PEEP level during surgery, were reported by Simon et al. [23].

#### Effects of anaesthesia, surgical position and pneumoperitoneum

**Fig. 3:**
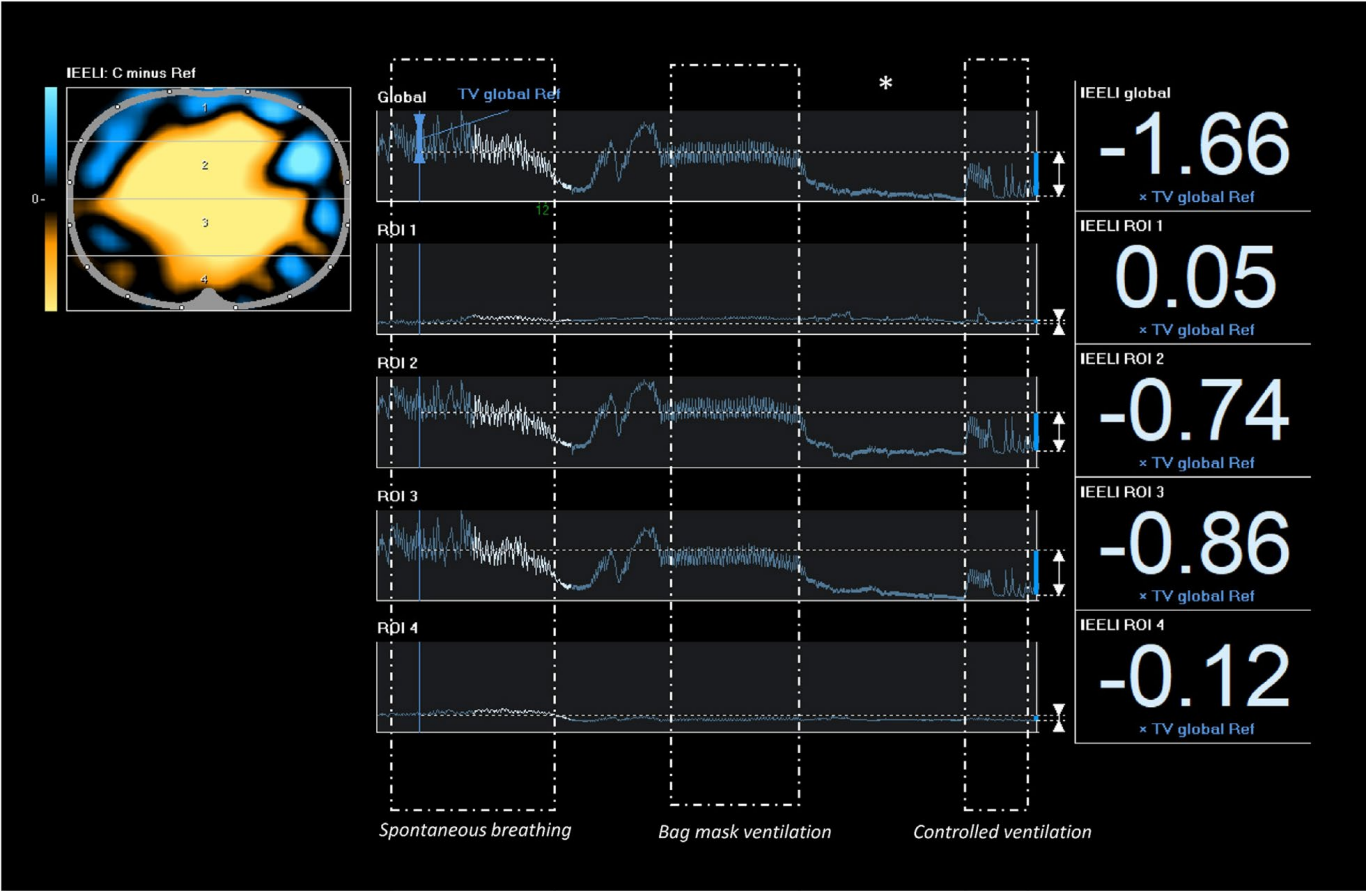
EIT findings during anaesthesia induction. Evolution of end-expiratory lung impedance (EELI) during different phases of general anaesthesia induction in an obese patient undergoing laparoscopic surgery. Functional EIT image (left) presents the regions with reduced EELI compared to the spontaneous breathing phase in orange colour. Global and regional EIT waveforms in four regions-of-interest (ROI) show the instantaneous regional changes in tidal impedance variation (TV) and end-expiratory impedance (middle) during the whole measurement period. Normalised changes in EELI between the initial phase and the onset of controlled ventilation are given in the right panel. * Significant difference at *p* ≤ 0.05

General anaesthesia and start of mechanical ventilation have immediate effects on regional lung expansion and ventilation (Fig. 3). Nothofer et al. [16] have shown how anaesthesia induction in obese patients quickly causes the formation of dorsal atelectasis. Buonanno et al. [24] documented that ventilation distribution becomes highly unbalanced in robotic laparoscopic surgery in Trendelenburg position. In these contexts, personalised PEEP with EIT allows the extent of these alterations to be limited. Surgical positioning (supine, lateral, prone) also significantly affects ventilation distribution, as shown by Ukere et al. [15]. EIT enables real-time monitoring of these variations, facilitating targeted and real-time posture adjustments.

In thoracic surgery, EIT was applied to monitor single-lung ventilation, thereby increasing safety, especially in elderly and frail patients [25–27].

#### Assessment of lung recruitment

Rosà et al. [28] used EIT to evaluate the effect of the recruitment-to-inflation (*R*/*I*) ratio in patients undergoing abdominal surgery, demonstrating that *R*/*I* reflected individual recruitment potential, correlated with recruited volumes measured by both gas dilution and EIT. Patients with *R*/*I* > 0.40 showed greater benefit from higher PEEP, with a significant reduction in collapse, dynamic strain and dead space. This suggests that *R*/*I* can be integrated with EIT in the assessment of intraoperative recruitability, facilitating personalised ventilation.

### Postoperative applications and respiratory phenotyping

In the postoperative phase, EIT has proven useful for monitoring the persistence of ventilatory alterations and identifying patients at risk of PPCs. Iwata et al. analysed patients after major surgery and demonstrated that some of them had asymmetrical ventilation patterns and persistent hypoventilated areas [29]. They found three different phenotypes with different incidence of PPCs in a population of high-risk patients admitted routinely to the ICU after surgery. Patients with inhomogeneous ventilation, either mostly ventral (phenotype 1) or dorsal (phenotype 3), showed higher incidence of PPCs and delayed ventilator weaning. These alterations were missed by conventional radiographic imaging at ICU entrance.

Another study [30] has linked postoperative ventilatory heterogeneity with an increase in pneumonia, respiratory failure and prolonged hospitalisation. In this context, EIT could provide prognostic parameters, and guide early interventions (respiratory physiotherapy, supplemental ventilatory support). This supports the EIT application for functional phenotyping [31] beyond intraoperative guidance.

### Other applications outside the operating room

EIT was applied in the nonoperating room anaesthesia during radiological or endoscopic procedures. EIT could identify lung volume reduction after the placement of one-way endobronchial valves in an animal model [32] and to evaluate the impact of alternative oxygenation/ventilation procedures on regional ventilation in bronchoscopic procedures [33]. Finally, the impact of deep sedation on regional ventilation has been explored especially in children undergoing diagnostic procedures (see below).

## Clinical applications in adult intensive care

Despite the increased interest in EIT, widespread adoption of EIT for personalisation of ventilator settings/strategies at the bedside is still in an early stage. Through a survey and focus-group interviews, we recently gathered insights in the clinicians’ experiences and perceived role of EIT in order to improve bedside implementation [34]. The main use-case of EIT currently is individualisation of PEEP setting, and although the technique is considered relevant and helpful, a lack of evidence-based guidelines was mentioned as the main reason that hampers bedside use. The recently published recommendations [1] may promote standardised use and processing/analysis of EIT data. We here update on a selection of clinical applications.

### PEEP setting

Recent meta-analyses on EIT-guided PEEP setting in ARDS described better respiratory mechanics as compared to conventional methods, and a potential for improved survival [35–37]. These results were not reproduced in a meta-analysis that combined ARDS and surgical patients, where patients undergoing anaesthesia showed better mechanics with an EIT strategy [38]. Evidence from prospective clinical studies powered on outcomes is lacking. Pilot studies are ongoing and Costa et al. [39] demonstrated the feasibility of an EIT-guided approach within a lung-protective ventilation strategy, where PEEP setting was based on the OD–CL method. Standardisation and homogenisation of the PEEP titration strategy is the key to the design of large-scale RCTs [1], as many techniques for PEEP setting with EIT exist [40, 41].

Selecting the right population for such approach is crucial. EIT-based PEEP titration was found reproducible in patients with hypoxemic respiratory failure. Aydeniz et al. [42] compared COVID-19 with pre-pandemic patients who underwent a clinical EIT-based PEEP titration. Overall, regional lung dynamics were comparable but COVID-19 patients generally had higher percentages of overdistention; these differences disappeared when correcting for the PEEP level. In severe ARDS patients on venovenous ECMO, Coppens et al. [43] found that the *R*/*I* manoeuvre could help identifying patients that likely benefit from EIT-guided PEEP titration. In low-recruitable patients (*R*/*I* ratio < 0.34 in their cohort), a moderate PEEP (8–10 cmH_2_O) may suffice, whereas personalisation with EIT could be helpful in moderate/higher recruitable patients.

EIT-guided PEEP may not be relevant in neurocritical patients with healthy lungs. Spatenkova et al. [44] found neither differences in ventilation homogeneity nor haemodynamic or ventilatory parameters with EIT-based PEEP as compared to a standard 5 cmH_2_O PEEP. PEEP titration was limited to the range of 2–9 cmH_2_O and resulted in similar PEEP settings between groups.

As mentioned in the experimental applications section, clinicians should consider the AOP during PEEP selection. EIT can provide insight in AOPs during a low-flow insufflation [45] and Sun et al. [46] described that the AOP derived from the global EIT signal reflects the lowest opening pressure in the lung, whereas regional AOPs could be higher.

### APRV

In ARDS patients who were sedated and receiving neuromuscular blockade during invasive ventilation with airway pressure release ventilation (APRV), Pequignot et al. [47] used EIT to set *P*_high_ within the time-controlled adaptive ventilation (TCAV) protocol. They performed a TCAV *P*_high_ trial where *P*_high_ was lowered by 4 cmH_2_O for every 5 min. *T*_low_ was set to maintain a constant driving pressure, allowing the EIT computation of overdistension and collapse during the trial. *P*_high_ was then set at the OD–CL crossing point, analogous to a PEEP trial. This could be a novel application to gain insight into regional ventilation distribution at various settings and specially to prevent overdistension during controlled ventilation with APRV.

### Lung perfusion and $\dot{V}/Q$ matching

Gao et al. confirmed that bicarbonate could be a good alternative to hypertonic saline as contrast agent for lung perfusion assessment in ventilated patients [48]. Although saline caused a larger drop in impedance after injection, regional perfusion distribution showed high agreement.

Leali et al. [49] calibrated $\dot{V}/Q$ maps using minute ventilation and CO in ARDS patients on controlled ventilation. CO was derived from invasive measurements but also noninvasively obtained from EIT directly using the pulsatility signal, which showed close correlation. This highlights the potential utility of EIT in providing insights into the degree of shunt and dead space compensation. The same research group found that $\dot{V}/Q$ mismatch was associated with several plasma biomarkers of lung epithelial and endothelial dysfunction, as well with impaired mechanics [50], meaning that $\dot{V}/Q$ mismatch on EIT could be an indicator of ARDS severity.

### EIT during spontaneous breathing

EIT in patients with spontaneous breathing is challenging because of variable breathing patterns and movement artefacts [1]. Concepts and EIT-derived parameters from the controlled ventilation setting should therefore be translated to patients with spontaneous breathing with caution.

EIT has been used as potential method for PEEP selection in patients on assisted ventilation. Heines et al. [51] developed the regional peak flow method that is based on changes in regional flow rate (i.e., derivative of the impedance signal) and thought to reflect changes in regional compliance. Mauri et al. [52] performed the OD–CL approach, while using dynamic changes in *P*_L_ instead of airway driving pressure to estimate pixel compliance; this requires synchronised measures of *P*_L_ and EIT. Lower work of breathing was found with this approach as compared to using the PEEP-FiO_2_ table. It should be noted that measuring pixel compliance during spontaneous breathing is prone to error as driving pressures are not static and the diaphragm generates a pressure gradient.

At different PEEP settings early after spontaneous breathing, Bassi et al. [53] showed that pendelluft was present in about half of patients (*n* = 11/20), though in small amounts (median of 28 mL). Pendelluft was not directly associated with the applied PEEP but with the breathing effort and respiratory mechanics.

**Fig. 4:**
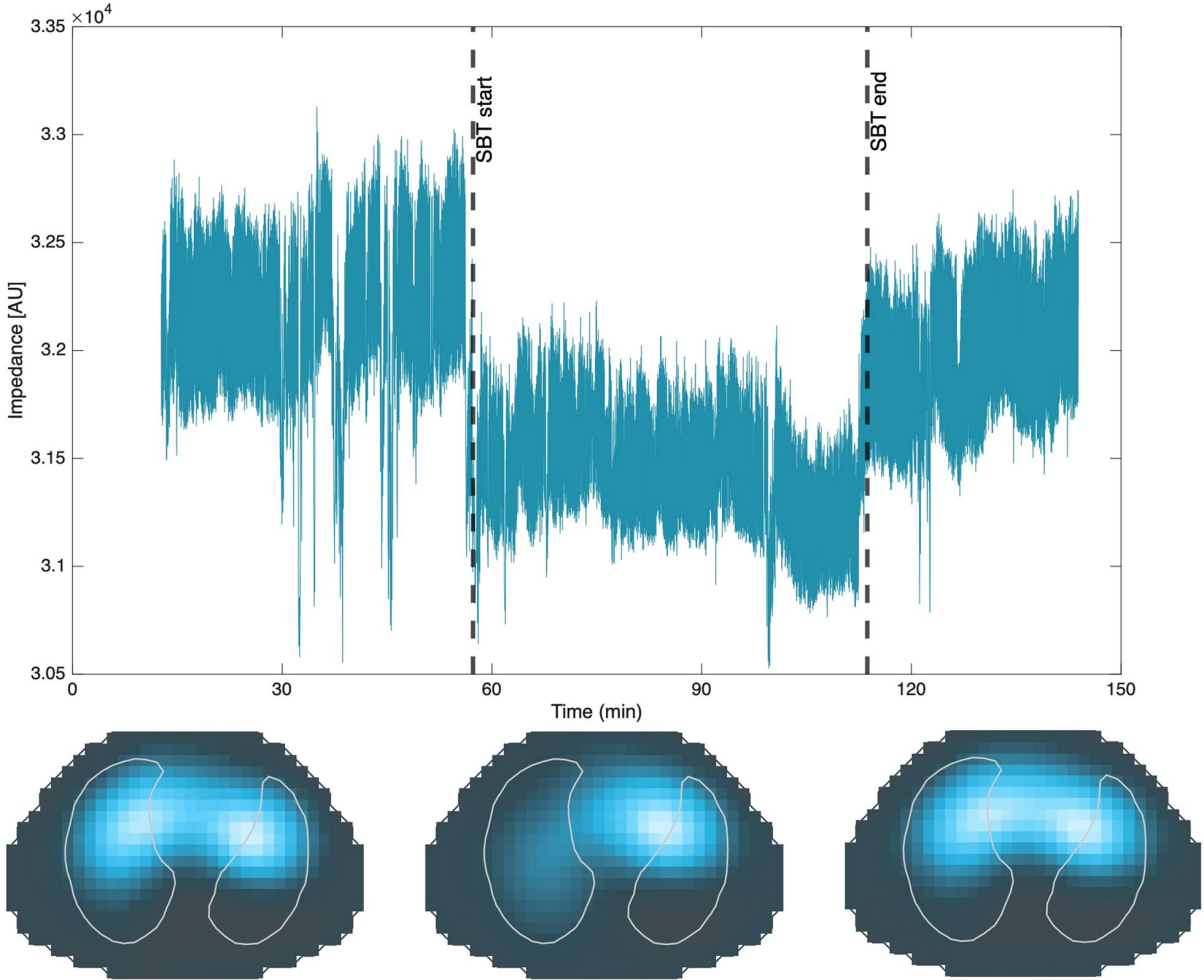
EIT examination of an adult ICU patient during a spontaneous breathing trial. Global EIT waveforms (top) and tidal impedance variation images (bottom) were recorded prior to, during, and after a spontaneous breathing trial (SBT) in an adult ICU patient. Note that end-expiratory lung impedance (EELI) is less stable during spontaneous breathing as compared to controlled ventilation, due to diaphragm contractions. During the start of the SBT, EELI drops due to the reduction in PEEP. Upon completion of the SBT and resuming mechanical ventilation, EELI increases again but not back to its initial value. Tidal impedance variation images show a different ventilation distribution during the SBT as compared to periods of mechanical ventilation. Representative case example kindly provided by Dr. Wisse, from her study [55]

Moving to the weaning phase, Phoophiboon et al. [54] found that ventrodorsal ventilation differences during a spontaneous breathing trial (SBT) could identify patients at risk for weaning failure. In line with these findings, Wisse et al. [55] described that end-expiratory lung impedance dropped after the start of the SBT and remained low after resuming mechanical ventilation (Fig. 4). Only few patients failed the SBT (*n* = 5/24), who showed high lung inhomogeneity. Machine learning was applied by Wang et al. [56] to identify features in EIT images during the SBT in patients with prolonged mechanical ventilation. They found good performance of the XGBoost model to predict weaning failure with regional ventilation delay being the most relevant feature.

## Clinical applications in neonatal and paediatric patients

The use of EIT in neonatal and paediatric patients is less frequent than in adults but its potential benefits are high. First, lung imaging methods are less frequently applied in neonates, infants and children than in adults, specifically those that utilise ionising radiation, to reduce the harmful effects of radiation on immature body tissues. Besides, the extremely fragile group of preterm patients generally requires very careful and cautious handling with preference for bedside examination methods to limit the patient transport. Second, unintentionally aggressive ventilator therapy may induce iatrogenic and long-lasting side effects that change the pulmonary structure and function, induce permanent lung tissue damage and lead to bronchopulmonary dysplasia. Therefore, close patient monitoring at the bedside and individualised therapy are highly relevant in sick neonates, infants and children.

EIT exhibits several advantageous features that favour its application in the neonatal and paediatric patients, however, its implementation is challenging, especially in very preterm and preterm neonates. These challenges are related to the EIT technology and the patient characteristics. Miniature EIT electrode interfaces fitting on the very small chest and still securing adequate but gentle skin–electrode contact are needed. Since the breathing and heart rates are higher in neonates and children than in adults, higher temporal resolution of EIT devices is required in these patients. Only modern devices secure sufficiently high scan rates (~50 Hz) to allow reliable analysis of dynamic physiological and pathophysiological events.

Essential findings of the main neonatal and paediatric EIT studies are given below.

### Effect of sedation, anaesthesia, surgery and physical therapy

Chidini et al. studied the effects of sedation in spontaneously breathing paediatric patients [57]. Sedation increased the spatial and temporal heterogeneity of ventilation identified by EIT which could be reversed by noninvasive ventilation but not by continuous positive airway pressure (CPAP). Lauret et al. confirmed that only pressure-support ventilation was able to restore end-expiratory lung volume after inhalational induction of anaesthesia using EIT [58].

**Fig. 5:**
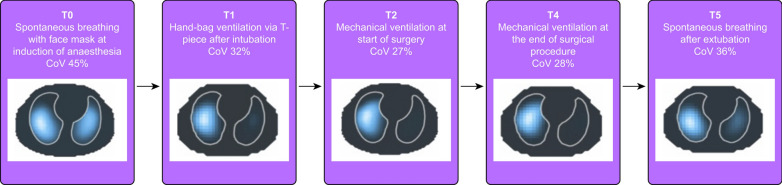
Functional EIT images of a 23-week old patient acquired at five perioperative time points. At T0, when patient was spontaneously breathing with face mask support, ventilation was relatively symmetrically distributed between the right and left lung regions. At T1, after intubation, an almost complete loss of ventilation in the left lung regions was observed that persisted at T2 and T4. (EIT recording at T3 was excluded during post hoc analysis because of insufficient data quality.) At T5, after extubation, there was a partial return of ventilation in the left lung, but not yet reaching the T0 pattern. The EIT findings obtained during this patient examination are consistent with potential right bronchus intubation that persisted during the whole 20-min surgery. CoV: ventrodorsal centre of ventilation. (Higher values imply more dorsal ventilation distribution.). The figure is reproduced under a Creative Commons Attribution 4.0 International License from [59]

In a small study on infants aged less than 1 year, Marchesini et al. analyzed the spatial distribution of ventilation at six perioperative timepoints, during induction of anaesthesia, after intubation, in course of surgery and after extubation [59]. The study identified the typical ventilation shift towards nondependent regions after intubation and during surgery, known also from other studies on adult patients [60] and children [61]. The striking finding of the study was the occurrence of one-sided intubation in three out of the 20 studied patients which went unnoticed during the anaesthesia but was identified during EIT data analyses (Fig. 5).

**Fig. 6:**
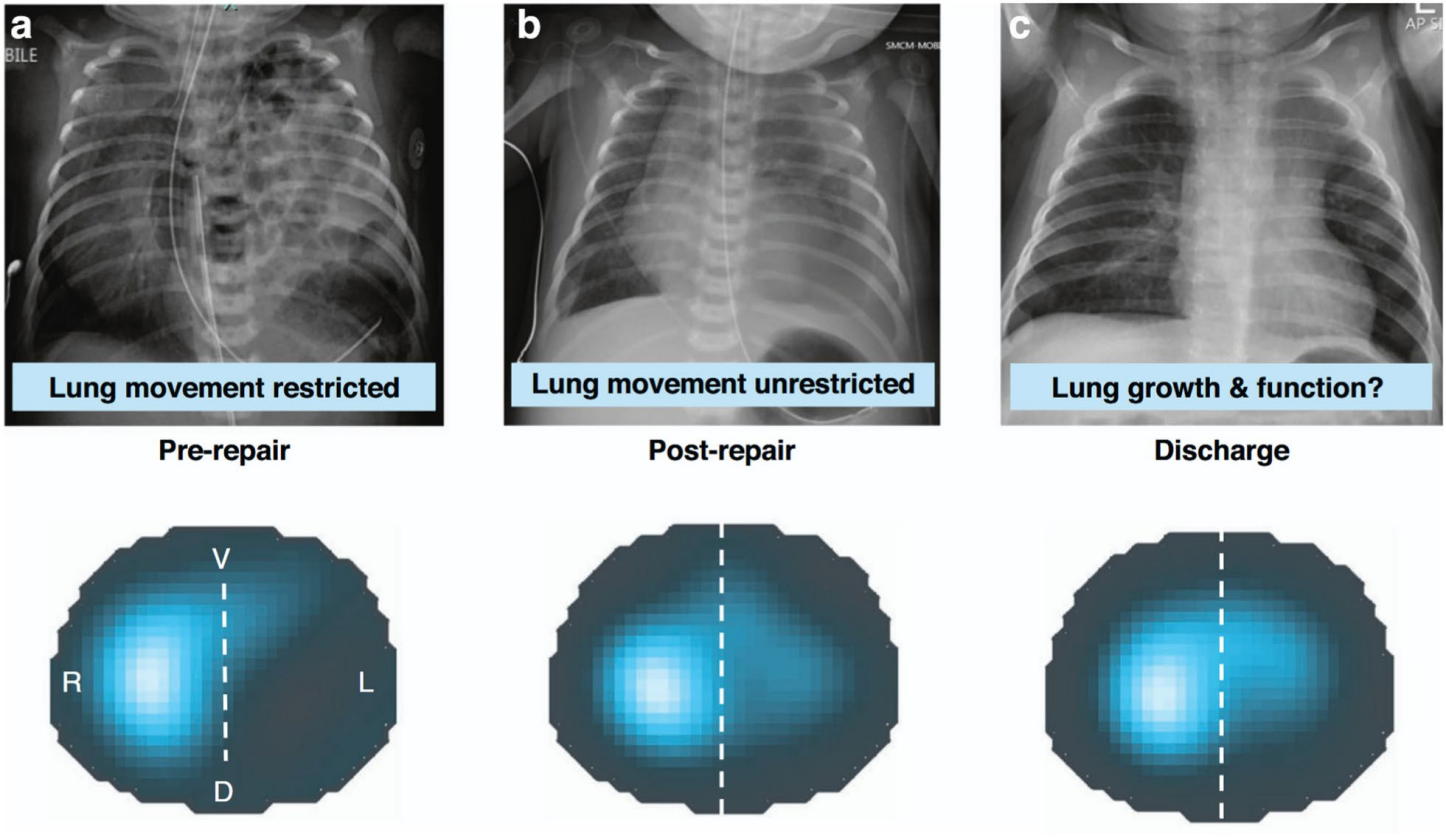
Chest radiography and functional EIT images acquired before and after the surgical repair of congenital diaphragmatic hernia. First lung imaging was performed before surgery (**a**). At this timepoint the left (ipsilateral) lung was hypoplastic and lung movement in both lungs (but especially the left) was restricted by hernia contents in thorax. Second lung imaging (**b**) was performed as soon as possible (defined by clinical stability) after the hernia repair. At this point the left lung was likely to be as equally hypoplastic as in pre-repair state, but movement was not restricted by herniated contents in thorax. Pre-discharge imaging (**c**) was undertaken at time clinical team commenced discharge planning. Magnitude of tidal ventilation in each lung region is expressed using a colour scale from dark blue (minimum) to light blue/white (maximum). R: right, L: left, V: ventral, D: dorsal (dotted line demarcates ipsilateral [left] and contralateral [right] hemithorax). The figure is reproduced under a Creative Commons Attribution 4.0 International License from [62]

The effect of surgical repair of congenital diaphragmatic hernia was studied by EIT by Douglas et al. [62]. The immediate postrepair changes in ventilation distribution could be identified along with the changes measured at the end of the NICU stay (Fig. 6).

The effects of active and passive chest physical therapy were assessed in paediatric patients by Moersdorf et al. [63]. Significant changes in ventilation distribution were only noted in patients with active therapy and in patients with localised radiological findings.

### Change in ventilator mode

Rahtu et al. analysed the continuously acquired EIT data in a group of preterm neonates during the time of transition from neurally adjusted ventilatory assist (NAVA) to time-cycled pressure-controlled ventilation (PCV) and vice versa [64]. No systematic changes in ventilation distribution between the two modes, regarding ventrodorsal or right-to-left distribution or the presence of ‘silent spaces’, were identified. The only difference between the two modes was the higher breathing pattern variability during NAVA, as expected.

In a larger study by Katheria et al., high-flow nasal cannula (HFNC) was compared with nasal CPAP [65]. EIT data revealed that infants who failed HFNC had a higher percentage of dependent ‘silent spaces’ compared with infants who tolerated HFNC well.

The study by Wisse et al. analysed the transition from invasive to noninvasive mechanical ventilation in preterm infants [66]. EIT data were evaluated 1 h before and after extubation. They showed higher degree of ventilation inhomogeneity in infants who failed extubation and significant reduction of dependent ‘silent spaces’ in successfully weaned patients.

Lozano-Ray et al. conducted a study on nonventilated children hospitalised with lower respiratory tract infection and in children without infection [67]. The authors modulated the respiratory system mechanics by placing a weighted blanket on the chest. They examined the ventilation distribution and measured oxygen saturation up to 30 min after blanket application and concluded the procedure was safe, well-tolerated without any major effects on ventilation distribution but with slight oxygenation improvement.

### PEEP setting

Application of EIT for individualisation of PEEP selection based on the OD–CL approach has also been shown to increase in children.

Wang et al. applied this method to determine individualised ‘optimum’ PEEP in a study on children undergoing laparoscopic hernia repair [68]. They analysed the incidence of atelectasis by lung ultrasonography directly after surgery and 1 h after extubation and compared the findings with a control group of children ventilated with a PEEP value of 5 cmH_2_O. EIT-based PEEP titration reduced the incidence and severity of postoperative atelectasis and led to less frequent oxygen desaturations.

Soltesz et al. acquired EIT data during a decremental PEEP trial in patients suffering from neonatal and paediatric ARDS [69]. During offline analysis, they showed that the clinically set PEEP differed from the EIT-derived PEEP balancing regional overdistention and collapse and concluded that EIT might contribute to better individualisation of PEEP. A similar conclusion was drawn by Lee et al. [70] who used the same EIT-based PEEP selection intraoperatively in supine and prone postures. The study demonstrated the need of higher PEEP in prone infants.

### Neonatal physiology

Janulionis et al. compared regional ventilation distribution in term neonates delivered by caesarean section and normal vaginal delivery [71]. The mode of delivery significantly affected early postnatal lung aeration, with larger fractions of lung areas being less ventilated in neonates delivered by caesarean section.

In a unique study, Gaertner et al. studied the breathing patterns in preterm infants immediately after birth [72]. During the first 10 min of life, EIT identified different patterns of lung ventilation and expansion development with distinct breath types of tidal breathing and breaths with holding or braking manoeuvres.

## Methodological studies

Following advancements in primary EIT image generation and data analyses were noted.

Rocheleau et al. developed an algorithm for real-time EIT image reconstruction using an infant CT scan database [73]. The algorithm was applied to EIT data of healthy and premature neonates. The EIT image quality of regional tidal ventilation and heartbeat-related impedance variation was improved by implementing prior anatomical information.

Digital signal filtering methods have been often applied during EIT data analyses to reduce the level of noise, drift and to separate the signal components associated with ventilation, and heartbeat-related phenomena. Wisse et al. compared three advanced filter techniques with conventional low-pass filtering [74], using patient data and simulated tracings. The new filters exhibited benefits according to the EIT parameter of interest, and outperformed the traditional low-pass filtering.

Francovich et al. [75] developed an approach for ventral and dorsal ROI selection in EIT data during controlled ventilation, with each ROI contributing equally to the sum of tidal impedance variations. This is similar to the established centre of ventilation parameter. The procedure can be extended to multiple layers as shown by Van Oosten et al. [76], allowing to study subtle changes in ventilation distribution.

## Conclusions

**Fig. 7:**
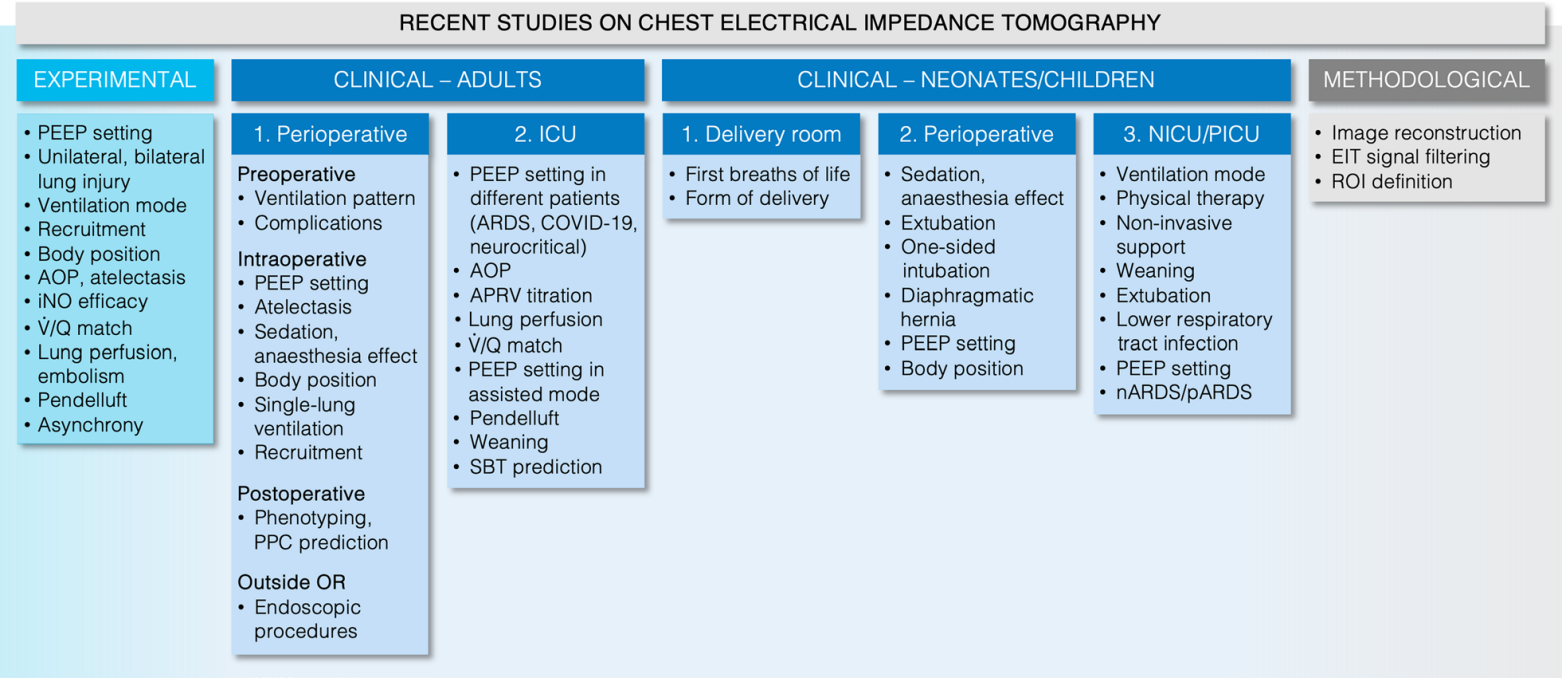
Summary of recent studies using chest EIT. Major effects and monitoring targets for the use of chest EIT in experimental, clinical and methodological studies reviewed in this article are presented. (The EIT use in spontaneously breathing pulmonology patients is not addressed since it was not included in this review for the intensive care medicine community.) ICU, intensive care unit; NICU, neonatal ICU; PICU, paediatric ICU; PEEP, positive end-expiratory pressure; AOP, airway opening pressure; iNO, inhaled nitric oxide; $\dot{V}$, ventilation; *Q*, perfusion; PPC, postoperative pulmonary complication; OR, operating room; ARDS, acute respiratory distress syndrome; APRV, airway pressure release ventilation; SBT, spontaneous breathing trial, nARDS, neonatal ARDS; pARDS, paediatric ARDS; ROI, region-of-interest

Our review with the summary of the most recent experimental and clinical research advances in chest EIT application (Fig. 7) is a clear evidence of the growing interest in this medical imaging method. The potential benefits of EIT use are being explored in a large variety of experimental and clinical scenarios but our review shows that the current major interest is linked with the use of EIT for individualised guiding the ventilator therapy. It focuses mainly on the PEEP setting using the generally available and automated procedure based on a decremental PEEP trial. This confirms the relevance of standardised procedures in EIT applications. However, further improvements are still needed (1) to exploit the full information content of EIT data (including lung perfusion), (2) to utilise intervention-free data or simple diagnostic interventions suitable for continuous assessment of regional lung function (i.e., less complex than a full decremental trial), (3) to combine EIT monitoring with findings of other clinically available methods, and (4) to implement decision-support systems. Since most of the available evidence on EIT use concerns physiological outcomes, conduction of controlled clinical studies demonstrating benefits on relevant clinical outcomes such as PPCs, length of stay, or hospital mortality is encouraged.

## Data Availability

Not applicable.

## Take home message

Chest electrical impedance tomography (EIT) offers noninvasive continuous assessment of regional lung and cardiovascular function at the bedside. Both experimental and clinical EIT research has intensified in the recent years demonstrating the benefits of EIT monitoring mainly in mechanically ventilated patients by allowing individually tailored ventilator therapy.

## References

[1] Scaramuzzo G, Pavlovsky B, Adler A, Baccinelli W, Bodor DL, Damiani LF, Franchineau G, Francovich J, Frerichs I, Giralt JAS, Grychtol B, He H, Katira BH, Koopman AA, Leonhardt S, Menga LS, Mousa A, Pellegrini M, Piraino T, Priani P, Somhorst P, Spinelli E, Handel C, Suarez-Sipmann F, Wisse JJ, Becher T, Jonkman AH (2024) Electrical impedance tomography monitoring in adult ICU patients: state-of-the-art, recommendations for standardized acquisition, processing, and clinical use, and future directions. Crit Care 28:377 39563476 10.1186/s13054-024-05173-x PMC11577873

[2] Frerichs I, Amato MB, van Kaam AH, Tingay DG, Zhao Z, Grychtol B, Bodenstein M, Gagnon H, Bohm SH, Teschner E, Stenqvist O, Mauri T, Torsani V, Camporota L, Schibler A, Wolf GK, Gommers D, Leonhardt S, Adler A (2017) Chest electrical impedance tomography examination, data analysis, terminology, clinical use and recommendations: consensus statement of the TRanslational EIT developmeNt stuDy group. Thorax 72:83–93 27596161 10.1136/thoraxjnl-2016-208357 PMC5329047

[3] Sousa MLA, Katira BH, Bouch S, Hsing V, Engelberts D, Amato MBP, Post M, Brochard LJ (2024) Limiting overdistention or collapse when mechanically ventilating injured lungs: a randomized study in a porcine model. Am J Respir Crit Care Med 209:1441–1452 38354065 10.1164/rccm.202310-1895OC

[4] Sousa MLA, Menga LS, Schreiber A, Docci M, Vieira F, Katira BH, Pellegrini M, Dubo S, Doufle G, Costa ELV, Post M, Amato MBP, Brochard L (2025) Individualized PEEP can improve both pulmonary hemodynamics and lung function in acute lung injury. Crit Care 29:107 40065461 10.1186/s13054-025-05325-7 PMC11892255

[5] Vivona L, Huhle R, Braune A, Scharffenberg M, Wittenstein J, Kiss T, Kircher M, Herzog P, Herzog M, Millone M, Gama de Abreu M, Bluth T (2023) Variable ventilation versus stepwise lung recruitment manoeuvres for lung recruitment: a comparative study in an experimental model of atelectasis. Eur J Anaesthesiol 40:501–510 36809307 10.1097/EJA.0000000000001808

[6] Mlček M, Borges JB, Otáhal M, Alcala GC, Hladík D, Kuriščák E, Tejkl L, Amato M, Kittnar O (2023) Real-time effects of lateral positioning on regional ventilation and perfusion in an experimental model of acute respiratory distress syndrome. Front Physiol 14:1113568 37020459 10.3389/fphys.2023.1113568 PMC10067565

[7] Pulletz S, Adler A, Kott M, Elke G, Gawelczyk B, Schadler D, Zick G, Weiler N, Frerichs I (2012) Regional lung opening and closing pressures in patients with acute lung injury. J Crit Care 27:323.e11–8 22033052 10.1016/j.jcrc.2011.09.002

[8] Pellegrini M, Sousa MLA, Dubo S, Menga LS, Hsing V, Post M, Brochard LJ (2024) Impact of airway closure and lung collapse on inhaled nitric oxide effect in acute lung injury: an experimental study. Ann Intensive Care 14:149 39312044 10.1186/s13613-024-01378-z PMC11420414

[9] Li J, Zhu M, Guo Y, Li W, He Q, Wang Y, Liu Y, Liu B, Liu Y, Wang W, Ji Z, Shi X (2025) Dynamic EIT technology for real-time non-invasive monitoring of acute pulmonary embolism: a porcine model experiment. Respir Res 26:7 39780173 10.1186/s12931-024-03090-9 PMC11715541

[10] Perier F, Tuffet S, Maraffi T, Alcala G, Victor M, Haudebourg AF, De Prost N, Amato M, Carteaux G, Mekontso Dessap A (2020) Effect of positive end-expiratory pressure and proning on ventilation and perfusion in COVID-19 acute respiratory distress syndrome. Am J Respir Crit Care Med 202:1713–1717 33075235 10.1164/rccm.202008-3058LE PMC7737587

[11] Han T, Qin Y, Zhao Z, Yang B, Liu X, Li L, Wei Z, Wei L, Liu Y, Fu F (2025) Calibration of ventilation/perfusion match in electrical impedance tomography: a novel method based on arterial blood pressure. Front Physiol 16:1545652 40182692 10.3389/fphys.2025.1545652 PMC11966062

[12] Bachmann MC, Cruces P, Diaz F, Oviedo V, Goich M, Fuenzalida J, Damiani LF, Basoalto R, Jalil Y, Carpio D, Hamidi Vadeghani N, Cornejo R, Rovegno M, Bugedo G, Bruhn A, Retamal J (2022) Spontaneous breathing promotes lung injury in an experimental model of alveolar collapse. Sci Rep 12:12648 35879511 10.1038/s41598-022-16446-2 PMC9310356

[13] Yoshida T, Torsani V, Gomes S, De Santis RR, Beraldo MA, Costa EL, Tucci MR, Zin WA, Kavanagh BP, Amato MB (2013) Spontaneous effort causes occult pendelluft during mechanical ventilation. Am J Respir Crit Care Med 188:1420–1427 24199628 10.1164/rccm.201303-0539OC

[14] Wittenstein J, Huhle R, Leiderman M, Mobius M, Braune A, Tauer S, Herzog P, Barana G, de Ferrari A, Corona A, Bluth T, Kiss T, Guldner A, Schultz MJ, Rocco PRM, Pelosi P, Gama de Abreu M, Scharffenberg M (2023) Effect of patient-ventilator asynchrony on lung and diaphragmatic injury in experimental acute respiratory distress syndrome in a porcine model. Br J Anaesth 130:e169–e178 34895719 10.1016/j.bja.2021.10.037

[15] Ukere A, März A, Wodack KH, Trepte CJ, Haese A, Waldmann AD, Böhm SH, Reuter DA (2016) Perioperative assessment of regional ventilation during changing body positions and ventilation conditions by electrical impedance tomography. Br J Anaesth 117:228–235 27440635 10.1093/bja/aew188

[16] Nothofer S, Steckler A, Lange M, Hezel A, Dumps C, Wrigge H, Simon P, Girrbach F (2024) Electrical impedance tomography-based evaluation of anesthesia-induced development of atelectasis in obese patients. J Clin Med 13:7736 39768660 10.3390/jcm13247736 PMC11678054

[17] Yang L, Lin Q, Zhao Z, Zhang J (2025) Early detection of pulmonary aspiration during anesthetic induction via bedside electrical impedance tomography monitoring: a case report. JVA Adv 2:100137

[18] Costa EL, Borges JB, Melo A, Suarez-Sipmann F, Toufen C Jr, Bohm SH, Amato MB (2009) Bedside estimation of recruitable alveolar collapse and hyperdistension by electrical impedance tomography. Intensive Care Med 35:1132–1137 19255741 10.1007/s00134-009-1447-y

[19] Scaramuzzo G, Priani P, Ferrara P, Verri M, Montanaro F, La Rosa R, Cammarota G, Volta CA, Spadaro S (2025) Longitudinal changes of electrical impedance tomography-based best PEEP in obese patients undergoing laparoscopic surgery: a prospective physiological study. Anaesth Crit Care Pain Med 44:101569 40518045 10.1016/j.accpm.2025.101569

[20] Pereira SM, Tucci MR, Morais CCA, Simoes CM, Tonelotto BFF, Pompeo MS, Kay FU, Pelosi P, Vieira JE, Amato MBP (2018) Individual positive end-expiratory pressure settings optimize intraoperative mechanical ventilation and reduce postoperative atelectasis. Anesthesiology 129:1070–1081 30260897 10.1097/ALN.0000000000002435

[21] Ma X, Fu Y, Piao X, De Santis Santiago RR, Ma L, Guo Y, Fu Q, Mi W, Berra L, Zhang C (2023) Individualised positive end-expiratory pressure titrated intra-operatively by electrical impedance tomography optimises pulmonary mechanics and reduces postoperative atelectasis: a randomised controlled trial. Eur J Anaesthesiol 40:805–816 37789753 10.1097/EJA.0000000000001901

[22] Pan L, Wu X, Gao L, Zhao Z, Yang L, Zhang J (2025) Intraoperative titration of positive end-expiratory pressure in urological patients undergoing laparoscopic procedures under lateral position: a randomized controlled trial. BMC Anesthesiol 25:311 40597668 10.1186/s12871-025-03171-2 PMC12210696

[23] Simon P, Girrbach F, Petroff D, Schliewe N, Hempel G, Lange M, Bluth T, Gama de Abreu M, Beda A, Schultz MJ, Pelosi P, Reske AW, Wrigge H, Network* PIotPV, the Clinical Trial Network of the European Society of A (2021) Individualized versus fixed positive end-expiratory pressure for intraoperative mechanical ventilation in obese patients: a secondary analysis. Anesthesiology 134:887–900 33843980 10.1097/ALN.0000000000003762

[24] Buonanno P, Marra A, Iacovazzo C, Merola R, De Siena AU, Servillo G, Vargas M (2023) Electric impedance tomography and protective mechanical ventilation in elective robotic-assisted laparoscopy surgery with steep Trendelenburg position: a randomized controlled study. Sci Rep 13:2753 36797394 10.1038/s41598-023-29860-x PMC9935531

[25] Tucci MR, Pereira SM, Costa ELV, Vieira JE (2020) Mechanical ventilation during thoracic surgery: towards individualized medicine. Ann Transl Med 8:842 32793686 10.21037/atm-20-2005 PMC7396770

[26] Liu K, Huang C, Xu M, Wu J, Frerichs I, Moeller K, Zhao Z (2019) PEEP guided by electrical impedance tomography during one-lung ventilation in elderly patients undergoing thoracoscopic surgery. Ann Transl Med 7:757 32042773 10.21037/atm.2019.11.95 PMC6989968

[27] Zha J, Yu YJ, Li GR, Wang SC, Qiao SG, Wang C, Bo HL (2023) Lung protection effect of EIT-based individualized protective ventilation strategy in patients with partial pulmonary resection. Eur Rev Med Pharmacol Sci 27:5459–5467 37401282 10.26355/eurrev_202306_32782

[28] Rosà T, Menga LS, Mastropietro C, Settanni D, Russo A, Frassanito L, Cascarano L, Catarci S, Delle Cese L, Zanfini BA, Scaramuzzo G, Dell’Anna AM, Spadaro S, Antonelli M, Grieco DL (2025) Evaluation of the potential for lung recruitment with the recruitment-to-inflation ratio during general anesthesia. Anesthesiology 143:1211–1224 40690301 10.1097/ALN.0000000000005677

[29] Iwata H, Yoshida T, Hoshino T, Aiyama Y, Maezawa T, Hashimoto H, Koyama Y, Yamada T, Fujino Y (2024) Electrical impedance tomography-based ventilation patterns in patients after major surgery. Am J Respir Crit Care Med 209:1328–1337 38346178 10.1164/rccm.202309-1658OC

[30] Jiang L, Deng Y, Xu F, Qiao S, Wang C (2024) Individualized PEEP guided by EIT in patients undergoing general anesthesia: a systematic review and meta-analysis. J Clin Anesth 94:111397 38278058 10.1016/j.jclinane.2024.111397

[31] Savino S, Gaetano S (2024) Functional phenotyping: a new role for electrical impedance tomography. Am J Respir Crit Care Med 209:1291–1292 38457807 10.1164/rccm.202402-0328ED PMC11146560

[32] Torsani V, Cardoso PFG, Borges JB, Gomes S, Moriya HT, Cruz AFD, Santiago RRS, Nagao CK, Fitipaldi MF, Beraldo MDA, Junior MHV, Mlcek M, Pego-Fernandes PM, Amato MBP (2024) First real-time imaging of bronchoscopic lung volume reduction by electrical impedance tomography. Respir Res 25:264 38965590 10.1186/s12931-024-02877-0 PMC11225379

[33] Gu Y, Zhang X, Li X, Min K, Deng H, Feng D, Zhou H, Wei J, Lv X (2025) Evaluating lung ventilation via electrical impedance tomography during flexible bronchoscopy with supraglottic jet ventilation: a prospective pilot study. J Anesth. 10.1007/s00540-025-03568-w 40864273 10.1007/s00540-025-03568-w

[34] Wisse JJ, Scaramuzzo G, Pellegrini M, Heunks L, Piraino T, Somhorst P, Brochard L, Mauri T, Ista E, Jonkman AH (2024) Clinical implementation of advanced respiratory monitoring with esophageal pressure and electrical impedance tomography: results from an international survey and focus group discussion. Intensive Care Med Exp 12:93 39432136 10.1186/s40635-024-00686-9 PMC11493933

[35] Sanchez-Piedra C, Rodriguez-Ortiz-de-Salazar B, Roca O, Prado-Galbarro FJ, Perestelo-Perez L, Sanchez-Gomez LM (2025) Electrical impedance tomography for PEEP titration in ARDS patients: a systematic review and meta-analysis. J Clin Monit Comput 39:987–997 40011398 10.1007/s10877-025-01266-2 PMC12474599

[36] Songsangvorn N, Xu Y, Lu C, Rotstein O, Brochard L, Slutsky AS, Burns KEA, Zhang H (2024) Electrical impedance tomography-guided positive end-expiratory pressure titration in ARDS: a systematic review and meta-analysis. Intensive Care Med 50:617–631 38512400 10.1007/s00134-024-07362-2 PMC11078723

[37] Sarkar S, Yalla B, Khanna P, Baishya M (2024) Is EIT-guided positive end-expiratory pressure titration for optimizing PEEP in ARDS the white elephant in the room? A systematic review with meta-analysis and trial sequential analysis. J Clin Monit Comput 38:873–883 38619718 10.1007/s10877-024-01158-x

[38] Gao Y, He H, Chi Y, Frerichs I, Long Y, Zhao Z (2024) Electrical impedance tomography guided positive end-expiratory pressure titration in critically ill and surgical adult patients: a systematic review and meta-analysis. BMC Pulm Med 24:582 39580405 10.1186/s12890-024-03394-y PMC11585246

[39] Costa ELV, Alcala GC, Tucci MR, Goligher E, Morais CC, Dianti J, Nakamura MAP, Oliveira LB, Pereira SM, Toufen C Jr, Barbas CSV, Carvalho CRR, Amato MBP (2024) Impact of extended lung protection during mechanical ventilation on lung recovery in patients with COVID-19 ARDS: a phase II randomized controlled trial. Ann Intensive Care 14:85 38849605 10.1186/s13613-024-01297-z PMC11161454

[40] Frerichs I, Schädler D, Becher T (2024) Setting positive end-expiratory pressure by using electrical impedance tomography. Curr Opin Crit Care 30:43–52 38085866 10.1097/MCC.0000000000001117

[41] Francovich JE, Katira BH, Jonkman AH (2025) Electrical impedance tomography to set positive end-expiratory pressure. Curr Opin Crit Care 31:319–327 39976222 10.1097/MCC.0000000000001255 PMC12052045

[42] Aydeniz E, Heines SJH, van Rosmalen F, van der Horst ICC, van Bussel BCT, Bergmans D (2025) Comparison of regional lung mechanics using electrical impedance tomography in mechanically ventilated COVID-19 vs. pre-pandemic patients: a retrospective study. Respir Med 247:108286 40752630 10.1016/j.rmed.2025.108286

[43] Coppens A, Aissi James S, Roze H, Juvin C, Repusseau B, Lebreton G, Luyt CE, Hekimian G, Chommeloux J, Pineton de Chambrun M, Combes A, Franchineau G, Schmidt M (2025) Optimum electrical impedance tomography-based PEEP and recruitment-to-inflation ratio in patients with severe ARDS on venovenous ECMO. Crit Care 29:195 40380232 10.1186/s13054-025-05437-0 PMC12084998

[44] Spatenkova V, Mlcek M, Mejstrik A, Cisar L, Kuriscak E (2024) Standard versus individualised positive end-expiratory pressure (PEEP) compared by electrical impedance tomography in neurocritical care: a pilot prospective single centre study. Intensive Care Med Exp 12:67 39103646 10.1186/s40635-024-00654-3 PMC11300775

[45] Louis B, Cour M, Argaud L, Guerin C (2024) The impact of PEEP on ventilation distribution in ARDS. Respir Care 69:1231–1238 39013571 10.4187/respcare.11569 PMC11469017

[46] Sun N, Brault C, Rodrigues A, Ko M, Vieira F, Phoophiboon V, Slama M, Chen L, Brochard L (2025) Distribution of airway pressure opening in the lungs measured with electrical impedance tomography (POET): a prospective physiological study. Crit Care 29:28 39819779 10.1186/s13054-025-05264-3 PMC11740639

[47] Pequignot B, Lescroart M, Levy B, Kimounn A, Koszutski M (2025) Electrical impedance tomography to set high pressure in time-controlled adaptive ventilation. J Crit Care 87:155033 39904168 10.1016/j.jcrc.2025.155033

[48] Gao Y, He Y, Chi Y, Yuan S, Wu S, Long Y, Zhao Z, He H (2025) Comparison of 5% sodium bicarbonate and 10% sodium chloride as contrast agents for lung perfusion with electrical impedance tomography: a prospective clinical study. BMC Pulm Med 25:190 40269841 10.1186/s12890-025-03665-2 PMC12016440

[49] Leali M, Marongiu I, Spinelli E, Chiavieri V, Perez J, Panigada M, Grasselli G, Mauri T (2024) Absolute values of regional ventilation-perfusion mismatch in patients with ARDS monitored by electrical impedance tomography and the role of dead space and shunt compensation. Crit Care 28:241 39010228 10.1186/s13054-024-05033-8 PMC11251389

[50] Spinelli E, Perez J, Chiavieri V, Leali M, Mansour N, Madotto F, Rosso L, Panigada M, Grasselli G, Vaira V, Mauri T (2025) Pathophysiological markers of acute respiratory distress syndrome severity are correlated with ventilation-perfusion mismatch measured by electrical impedance tomography. Crit Care Med 53:e42–e53 39445936 10.1097/CCM.0000000000006458

[51] Heines SJH, de Jongh SAM, de Jongh FHC, Segers RPJ, Gilissen KMH, van der Horst ICC, van Bussel BCT, Bergmans D (2025) A novel positive end-expiratory pressure titration using electrical impedance tomography in spontaneously breathing acute respiratory distress syndrome patients on mechanical ventilation: an observational study from the MaastrICCht cohort. J Clin Monit Comput 39:127–139 39196479 10.1007/s10877-024-01212-8 PMC11821668

[52] Mauri T, Grieco DL, Spinelli E, Leali M, Perez J, Chiavieri V, Rosa T, Ferrara P, Scaramuzzo G, Antonelli M, Spadaro S, Grasselli G (2024) Personalized positive end-expiratory pressure in spontaneously breathing patients with acute respiratory distress syndrome by simultaneous electrical impedance tomography and transpulmonary pressure monitoring: a randomized crossover trial. Intensive Care Med 50:2125–2137 39527121 10.1007/s00134-024-07695-y PMC11588931

[53] Bassi T, Dianti J, Roman-Sarita G, Bellissimo C, Morris IS, Slutsky AS, Brochard L, Ferguson ND, Zhao Z, Yoshida T, Goligher EC (2025) Effect of higher or lower PEEP on pendelluft during spontaneous breathing efforts in acute hypoxemic respiratory failure. Respir Care 70:126–133 39964850 10.1089/respcare.12193

[54] Phoophiboon V, Rodrigues A, Vieira F, Ko M, Madotto F, Schreiber A, Sun N, Sousa MLA, Docci M, Brault C, Menga LS, Telias I, Piraino T, Goligher EC, Brochard L (2025) Ventilation distribution during spontaneous breathing trials predicts liberation from mechanical ventilation: the VISION study. Crit Care 29:11 39773268 10.1186/s13054-024-05243-0 PMC11705700

[55] Wisse JJ, Goos TG, Jonkman AH, Somhorst P, Reiss IKM, Endeman H, Gommers D (2024) Electrical impedance tomography as a monitoring tool during weaning from mechanical ventilation: an observational study during the spontaneous breathing trial. Respir Res 25:179 38664685 10.1186/s12931-024-02801-6 PMC11044327

[56] Wang P, Chen TH, Chang MY, Hsia HY, Dai M, Liu Y, Hsu YL, Fu F, Zhao Z (2025) An explainable artificial intelligence framework for weaning outcomes prediction using features from electrical impedance tomography. Comput Methods Programs Biomed 267:108811 40339409 10.1016/j.cmpb.2025.108811

[57] Chidini G, Marchesi T, Catenacci SS, Florio G, Conti G, Lanni S, Filocamo G, Patria F, Guerrini M, Milani G, Grasselli G (2025) Effects of noninvasive respiratory support on ventilation distribution during spontaneous breathing sedation in preschool/school-aged children: an electrical impedance tomography study. Paediatr Anaesth 35:562-572 40119601 10.1111/pan.15098 PMC12149492

[58] Lauret V, Guerin C, Boussena S, De-Queiroz M, Bouvet L, Baudin F (2025) Pressure support ventilation improves ventilation during inhalational induction of anesthesia in children: a pilot study. J Clin Anesth 101:111710 39693684 10.1016/j.jclinane.2024.111710

[59] Marchesini V, Corlette S, Sheppard S, Davidson A, Tingay D (2024) Evaluation of lung homogeneity in neonates and small infants during general anaesthesia using electrical impedance tomography: a prospective observational study. BJA Open 12:100344 39364288 10.1016/j.bjao.2024.100344 PMC11447312

[60] Frerichs I, Hahn G, Golisch W, Kurpitz M, Burchardi H, Hellige G (1998) Monitoring perioperative changes in distribution of pulmonary ventilation by functional electrical impedance tomography. Acta Anaesthesiol Scand 42:721–726 9689281 10.1111/j.1399-6576.1998.tb05308.x

[61] Rosalba D, Meneghetti G, Verdina F, Solai C, Azzolina D, Petronio L, Guaraglia M, Buscaglia R, Saviolo G, Furlan G, Vietti F, Biasucci D, Spadaro S, Simonte R, De Robertis E, Longhini F, Penpa S, Ubertazzi M, Panuccio E, Aluffi P, De Cilla S, Brucoli M, Vaschetto R, Cammarota G (2025) Patterns of lung aeration assessed through electrical impedance tomography in paediatric patients undergoing elective surgery: insights from a prospective and observational data-registry. J Anesth Analg Crit Care 5:34 40551255 10.1186/s44158-025-00254-x PMC12183812

[62] Douglas E, Ferguson KN, Tingay DG (2025) Regional lung function in congenital diaphragmatic hernia assessed using electrical impedance tomography. Pediatr Res 98:1773-1779 40533505 10.1038/s41390-025-04185-9 PMC12602320

[63] Moersdorf J, Muders T, Putensen C, Heller U, Mueller A, Schroeder L (2025) Monitoring of regional ventilation distribution using electrical impedance tomography in pediatric patients with chest physiotherapy-a feasibility study. Pediatr Pulmonol 60:e71014 40008692 10.1002/ppul.71014 PMC11863537

[64] Rahtu M, Frerichs I, Becher TH, Pokka T, Waldmann AD, Papadouri T, van Kaam AH, Rimensberger PC, Bayford R, Peltoniemi O, Kallio M (2025) Changes in respiratory patterns from pressure control ventilation to neurally adjusted ventilatory assist assessed by electrical impedance tomography. Pediatr Pulmonol 60:e71077 40170630 10.1002/ppul.71077

[65] Katheria A, Ines F, Hough J, Rich W, Morales A, Sanjay S, Poeltler D, Finer N (2025) Changes in lung aeration with high-flow nasal cannula compared to nasal CPAP in preterm infants. J Perinatol 45:817–822 40122991 10.1038/s41372-025-02267-4

[66] Wisse JJ, Goos TG, Gommers D, Endeman H, Kroon AA, Reiss IKM, Jonkman AH (2025) Electrical impedance tomography during the extubation phase in very preterm born infants. Neonatology 122:366–375 40031894 10.1159/000544811 PMC12129417

[67] Lozano-Ray E, Argent AC, Lupton-Smith A, Morrow BM (2025) The effect of limiting anterior chest wall movement with a weighted blanket on comfort, oxygenation, and ventilation homogeneity in nonventilated infants and children admitted to hospital with lower respiratory tract infections: an exploratory pilot study. Pediatr Pulmonol 60:e71186 40637389 10.1002/ppul.71186 PMC12243703

[68] Wang SH, Wang Y, Li SY, Jiang L, Mao YF, Xia Q, Gan H (2025) Effect of individualized PEEP titrated by EIT on postoperative atelectasis in children undergoing laparoscopy: a randomized controlled trial. Int J Med Sci 22:3007–3013 40657398 10.7150/ijms.112280 PMC12243984

[69] Soltesz L, Leyens J, Vogel M, Muders T, Putensen C, Kipfmueller F, Dresbach T, Mueller A, Schroeder L (2025) EIT guided evaluation of regional ventilation distributions in neonatal and pediatric ARDS: a prospective feasibility study. Respir Res 26:60 39972380 10.1186/s12931-025-03134-8 PMC11841312

[70] Lee JH, Kang P, Park JB, Ji SH, Jang YE, Kim EH, Kim JT, Kim HS (2024) Determination of optimal positive end-expiratory pressure using electrical impedance tomography in infants under general anesthesia: comparison between supine and prone positions. Paediatr Anaesth 34:758–767 38693633 10.1111/pan.14914

[71] Janulionis A, Sutova V, Langiene V, Virsilas E, Drejeriene V, Liubsys A, Valiulis A (2025) Electrical impedance tomography confirmed the impact of the method of delivery of term neonates on early lung aeration. Adv Clin Exp Med 34:1105–1112 39868740 10.17219/acem/190742

[72] Gaertner VD, Büchler VL, Waldmann A, Bassler D, Rüegger CM (2024) Deciphering mechanisms of respiratory fetal-to-neonatal transition in very preterm infants. Am J Respir Crit Care Med 209:738–747 38032260 10.1164/rccm.202306-1021OC

[73] Rocheleau CJ, Overtonda TD, da Rosa Jr. NB, Saulnier GJ, Shishvan OR, Baker CD, Enzer KG, Mueller JL (2025) Use of an anatomical atlas in real-time EIT reconstructions of ventilation and pulsatile perfusion in preterm infants. Sci Rep 15:29622 40796647 10.1038/s41598-025-15543-2 PMC12343824

[74] Wisse JJ, Somhorst P, Behr J, van Nieuw Amerongen AR, Gommers D, Jonkman AH (2024) Improved filtering methods to suppress cardiovascular contamination in electrical impedance tomography recordings. Physiol Meas 45:055010 10.1088/1361-6579/ad46e3 38697210

[75] Francovich JE, Somhorst P, Gommers D, Endeman H, Jonkman AH (2024) Physiological definition for region of interest selection in electrical impedance tomography data: description and validation of a novel method. Physiol Meas 45:105002 10.1088/1361-6579/ad7f1f 39317238

[76] Van Oosten JP, Francovich JE, Somhorst P, van der Zee P, Endeman H, Gommers D, Jonkman AH (2024) Flow-controlled ventilation decreases mechanical power in postoperative ICU patients. Intensive Care Med Exp 12:30 38502268 10.1186/s40635-024-00616-9 PMC10951187

